# An Integrated Framework for Process-Driven Model Construction in Disease Ecology and Animal Health

**DOI:** 10.3389/fvets.2017.00155

**Published:** 2017-09-27

**Authors:** Rebecca Mancy, Patrick M. Brock, Rowland R. Kao

**Affiliations:** ^1^College of Veterinary, Medical and Life Sciences, Institute of Biodiversity, Animal Health and Comparative Medicine, University of Glasgow, Glasgow, United Kingdom; ^2^Boyd Orr Centre for Population and Ecosystem Health, University of Glasgow, Glasgow, United Kingdom

**Keywords:** process models, modelling, model construction, epidemiology, infectious disease, disease ecology, foot-and-mouth disease, bovine tuberculosis

## Abstract

Process models that focus on explicitly representing biological mechanisms are increasingly important in disease ecology and animal health research. However, the large number of process modelling approaches makes it difficult to decide which is most appropriate for a given disease system and research question. Here, we discuss different motivations for using process models and present an integrated conceptual analysis that can be used to guide the construction of infectious disease process models and comparisons between them. Our presentation complements existing work by clarifying the major differences between modelling approaches and their relationship with the biological characteristics of the epidemiological system. We first discuss distinct motivations for using process models in epidemiological research, identifying the key steps in model design and use associated with each. We then present a conceptual framework for guiding model construction and comparison, organised according to key aspects of epidemiological systems. Specifically, we discuss the number and type of disease states, whether to focus on individual hosts (e.g., cows) or groups of hosts (e.g., herds or farms), how space or host connectivity affect disease transmission, whether demographic and epidemiological processes are periodic or can occur at any time, and the extent to which stochasticity is important. We use foot-and-mouth disease and bovine tuberculosis in cattle to illustrate our discussion and support explanations of cases in which different models are used to address similar problems. The framework should help those constructing models to structure their approach to modelling decisions and facilitate comparisons between models in the literature.

## Background

The use of models is becoming increasingly popular for understanding the biological processes that drive the host-to-host spread and within-host progression of infectious diseases, for both theoretical and applied problems. Key epidemiological processes include transmission arising from contact between infectious and susceptible hosts, disease progression within hosts (e.g., onset of symptoms, recovery), and interventions such as vaccination or treatment. These processes are dynamic, and this time dimension can be captured through changes in the epidemiological state of individuals over time. Models in which these processes are represented explicitly, often referred to as *mechanistic* or *process models*,^1^ are increasingly common in disease ecology, including in veterinary epidemiology.

The explicit incorporation of biological mechanism makes process models ideal for studying systems in which population-level effects (such as disease outbreaks) arise from individual-level processes in ways that are difficult to anticipate (such as details of how infectious individuals today feed into generating new infections tomorrow) (1). By changing model inputs to simulate interventions, we can also use them to analyse the effects of policies that cannot be tested in the real world because doing so would be infeasible due to time or resource constraints or because such experiments would be ethically undesirable. Such effects would be difficult or impossible to investigate using standard statistical frameworks.

A wide range of options exists for constructing process models. Early epidemiological process models typically took the form of differential equation models representing susceptible-infectious-susceptible (SIS) or susceptible-infectious-removed (SIR) disease dynamics. However, increasingly, researchers are using models that incorporate higher levels of biological detail. One approach is to use agent-based models (ABMs; also referred to as individual-based models) in which each host is modelled explicitly and its state (i.e., its disease state plus all relevant epidemiological characteristics) progresses according to a set of rules. These rules can be simple, but can and often do incorporate greater complexity. Many more types of process model exist, including partial differential equation models and cellular automata. This diversity makes it challenging to make defensible decisions when developing new models, and complicates the task of elucidating any inconsistencies between studies, assessing the weight of the evidence for particular claims, and identifying research gaps. However, most overviews focus on describing technical aspects of each type and include only limited discussion of their relationship with biology, while original research articles typically compare only a small number of model types.

Here, we clarify the relationship between process modelling approaches both in relation to high-level motivations for their use and to lower-level decisions about the characteristics of a particular model. We describe five distinct motivations, identifying the key steps in model design and uses associated with each and present a framework that forms the conceptual basis for making modelling decisions, considering the constraints imposed by the epidemiological system, the available data, relevant knowledge and expertise, and the questions of interest. Our approach is explanatory rather than prescriptive, as the implications of modelling decisions are highly dependent on context. We anticipate that this analysis will be most valuable for researchers who are relatively new to process modelling, but believe it has broader value by providing an organisational structure for comparing and contrasting modelling approaches.

## From System to Model

A major preoccupation when choosing a modelling approach is that it should, in some sense, be “correct.” Although Box’s (2) claim that “all models are wrong but some are useful” has become something of a mantra, its practical implications remain a justifiable concern. Nearly a century earlier, Claude Bernard (3, 4) had noted that, like models, scientific theories are always “wrong” insofar as they are “only partial and provisional truths,” but had emphasised their necessary role in science as “steps on which we rest, so as to go on with investigation.” In evaluating models, Odenbaugh (5) argues for a shift of focus away from model “truth” towards the appropriateness of particular modelling “idealisations” (simplifications or abstractions), which should be considered in the context of the biological system to which we apply the model and the questions it helps us answer. Below, we discuss the application of these principles to epidemiological modelling.

To provide meaningful context, we discuss model construction decisions with reference to two illustrative^2^ disease systems in veterinary epidemiology involving cattle: foot-and-mouth disease (FMD; caused by FMD virus) and bovine tuberculosis (bTB; caused by *Mycobacterium bovis*). To keep the focus on the underlying concepts and avoid introducing multiple epidemiological systems, we limit practical examples discussed to those provided by modelling work on these two diseases. These two high profile pathogens have important animal health implications and are associated with a considerable body of research using process models, much of which has focused on recent UK epidemics: a major outbreak of FMD occurred in 2001 and was controlled by large-scale intervention (6), while bTB remains endemic in the UK (7). In thinking about potential influences of the epidemiology of bTB and FMD in cattle, we observe that: different numbers of cows are kept on farms of variable size; cows have fixed *attributes* (e.g., breed) and changing *states* (e.g., age); and there can be variation in the environment at, around, and between farms. We note that cattle come into contact with one another through activities such as grazing on the same or neighbouring pastureland, and are moved between farms as part of trade, slaughter, and breeding activities. These factors can affect epidemiological outcomes and it is important to consider whether and how to model them. For example, in an ABM, we would represent cattle hosts as distinct agents. The attributes, states, and movement in continuous space of these individuals could be simulated and tracked through time. The model would be initiated with a population of cattle and seeded with infection. When the model was run, the initial population would be subject to processes such as aging, movement, infection, and recovery. Cattle movement within farms could be modelled as a random process, while between-farm movement could be informed by trade volume data, and transmission between hosts determined by contact between susceptible and infectious individuals. As time progressed, the state of every host would be updated following the specified process rules.

Because the structure of detailed ABMs maps closely to our understanding of the real world, they often appear intuitive. However, they are time-consuming to construct because they involve making many decisions about the processes to model. Many alternatives exist, yet selection can be challenging. The remainder of this paper consists of two main sections: in the first, we distinguish between five motivations for using process models and the particular aspects of model construction to focus on for each; in the second, we provide an organisational structure for navigating modelling decisions. The first of these sections is more philosophical and the second more practically oriented; they can be read together or as stand-alone sections.

## The Questions We Wish to Answer

The appropriateness of a particular model depends on both our precise research question and our motivations for using a process model to help answer it. Clarifying our motivations for using a model and the steps required to use it in this way helps guide modelling decisions towards the aspects of the model that are most critical for the way it will be used and help us determine the appropriate level of complexity or output accuracy, something we return to in Section “Applying the Model Construction Approach.” Irrespectively of whether the focus is on highly specific or abstract systems, one or several motivations can apply in each piece of work. These are not always made explicit in the text of an article, but distinguishing between them can help us to understand the role of modelling within a piece of work and, therefore, evaluate its appropriateness. In this section, we present five motivations for using process models in epidemiology.^3^ In Table 1, we describe start and end points for each motivation, provide illustrative questions or observations associated with each (with references, where available for bTB/FMD and otherwise for more abstract disease systems), and identify the primary focus during model construction. In the main text, we provide an overview of the associated steps involved.

**Table 1:** Five categories of motivations for modelling, illustrative prompt questions, and focus during model construction.

	Motivations for modelling	Illustrative prompt questions or statements and references	Focus during model construction
*Mapping and formalising theory—providing conceptual frameworks*	Modelling helps provide a conceptual framework (for self or others)	What are the key entities and processes required to model bTB and how might we formulate them in the most conceptually useful way?	Conceptual clarity of key entities and processes and formalisation of these
	Begin with informal understanding or verbal theory; obtain a precise formal representation of the theory (a full model) or of concepts and subcomponents of it	Are there similar concepts in associated areas that could apply (e.g., how does reproductive potential relate to *R*_0_)? (9)	

*Exploring theory—exploring possibilities*	A model formalising theory is used to constrain relationships between entities so that system behaviours can be explored	Is infection invasion success dependent on spatial clustering? (10)	Accurate representation of relevant aspects of the theory in the model
	Begin with a model that formalises theory; obtain a set of possible behaviours given those processes	What is the probability of bTB persisting in cattle herds of different sizes? (11)	No explicit use of data is required

*Building theory—generating hypotheses and explanations*	The structure of the formalised model focuses our attention on particular processes and parameters, changes to which constitute testable hypotheses	Following the 10-year randomised badger culling trial, bTB incidence in cattle decreased in the badger culling area, but increased in adjoining areas (12)	Observing the way structures and parameters suggest model reformulations
	Begin with an observation or data; obtain precise hypotheses. NB: theory building often conducted iteratively with theory testing (below)	The 1967–1968 UK foot-and-mouth disease (FMD) epidemic was characterised by rapid early spread followed by slower later spread (13)	

*Testing theory—identifying mechanisms*	To generate empirically relevant and measurable predictions, for the purpose of falsification	Does the incorporation of transmission heterogeneity allow us to better explain the data? (14)	Incorporation of mechanisms into a model in ways that allow us to establish whether observed phenomena can be reproduced; structural equivalence between data and model outputs
	Begin with a model that encapsulates a theory; obtain predictions that can be compared with data to help pinpoint incorrect mechanisms		

*Applying theory—generating accurate predictions*	To make forecasts, predict responses under intervention, and examine counterfactual scenarios	How might FMD epidemiological dynamics have differed under alternative culling scenarios during the 2001 FMD outbreak? (15)	Ensuring key mechanisms are replicated as closely as required to accurately reproduce real-world phenomena and data
	Begin with a model that is assumed to be true; obtain hindcasts/forecasts, and predictions relating to counterfactuals and other systems	What difference might incursion location and speed of deployment make to the effectiveness of FMD reactive ring vaccination? (16)	

### Mapping and Formalising Theory

When our initial thinking about an epidemiological system is relatively imprecise, the process of formalising our ideas helps improve conceptual clarity and can even allow us to develop new epidemiological concepts or refine existing ones. By “mapping and formalising theory,” we refer to the process by which we go from our (typically informal) understanding of an epidemiological system derived from verbal theory or experience, to a formal model that can be written down (e.g., using mathematical symbols or computer code). Formal and symbolic models provide conceptual frameworks that allow us to reason about more complex systems than would be possible using purely verbal arguments. Odenbaugh (5) points out that “model building is first and foremost a *strategy* for coping with an extraordinarily complex world,” making this an important motivation for model construction. Heesterbeek (9) describes how formalising theory led to the development of the basic reproductive number (sometimes called “rate” or “ratio”), *R*_0_, that has become one of the most important concepts in contemporary infectious disease epidemiology. *R*_0_ is now widely used independently of the original models (or indeed of any model at all), as a communication tool, to raise and formulate new questions about epidemiological systems and how to model them.

Although the benefits of increased conceptual precision that arise from formalisation are often considered a side effect of model development (and rarely reported in research articles), formalising theory can be a deliberate strategy to help us understand complex systems. In this case, during model construction, we focus on questions about how best to represent the key processes in our system. These questions can relate to direct analysis of our biological system; alternatively, we may seek inspiration in similar concepts from other domains, attempting to identify the link between the two. If a mapping can be found, then concepts, insights, and results can be transferred—for example, identifying parallels with network models developed in the statistical physics literature has been particularly fruitful in epidemiology, e.g., Ref. (17)—but even failed attempts would ideally be reported because they help us identify inconsistencies, potentially leading to the development of new concepts.

### Exploring Theory

Once a theory has been incorporated into a formal model, its implications can be explored. An epidemiological model constrains the range of outcomes that can arise, so it can be used to deduce or simulate the range of possible system behaviours or the probability that those behaviours arise. This might be achieved in different ways, including mathematical deduction that allows us to develop and prove theorems and experimental approaches based on simulation. For example, process models could be used to explore the range of potential long-run behaviours of epidemiological systems (e.g., whether endemicity can arise), or to establish whether chaotic behaviour is possible (5).

When exploring theory, we begin with a model that represents that theory and use logical reasoning, mathematical deduction or computational approaches to understand the range of possible behaviours that can be generated under the assumptions of the theory, and potentially their associated probabilities. When used to explore theory, neither the model construction nor the exploration stage focuses directly on the correspondence between model and data, but rather on the correspondence between theory and model, to ensure that the implications for theory of model findings are clear. The theory under investigation may be general and abstract, and correspond to a hypothetical disease, or a specific epidemiological system. Exploration does not rely on the existence of data; even in the absence of data, it can guide epidemiological science by suggesting behaviours to look for in empirical work and their expected frequencies, and by helping to determine sample sizes (18).

### Building Theory

Theory building consists of generating possible explanations for empirical observations. When we use models to help us with this task, our motivation is to help us formulate hypotheses by suggesting potential causal mechanisms that explain an observed phenomenon. The phenomenon to explain could take the form of a general trend or pattern observed in a range of epidemiological systems, or an observation arising from a specific dataset. For example, following the 10-year randomised badger culling trial, bTB incidence in cattle decreased in the badger culling area, but increased in adjoining areas. This observation was initially counter-intuitive and theory building was required to explain how it arose (12).

When constructing models to assist us in theory building, our starting point is an unexplained observation. Our primary focus in their construction is on the way in which structures and parameters, usually from an existing model, might suggest reformulations or extensions that constitute hypotheses. These hypotheses can be based on mechanisms drawn from general theory (e.g., about different kinds of host contact structure) or system-specific mechanisms, such as those based on experiential knowledge of the system (e.g., differences between England and Scotland in cattle-trading behaviour). These mechanisms need to be incorporated into the model before proceeding with theory testing (see Testing Theory) when the model will be used to characterise their effect and make comparisons with empirical observations.

### Testing Theory

Theory testing refers to attempts to establish whether a theory provides a good explanation for empirical observations or data. Our motivation for using models for this purpose is their ability to generate falsifiable predictions that we can compare with existing data or observations or employ to guide data collection protocols or experiments. Although verbal theory is sometimes sufficient to make falsifiable predictions, a model can be valuable if we want to generate quantitative predictions or if the system is too complex to reason about otherwise. When using a model to test theory, no initial claim is made about the truth status of the model, and we often acknowledge that it is idealised or incomplete. Model predictions used to test theory are not forecasts; rather, the intention is that they are empirically falsifiable. Indeed, when models fail to make accurate predictions, they often fail for reasons that are very informative about the systems under study (5).

When testing theory, it is important to incorporate hypothesised mechanisms into the model in a way that allows us to establish whether it produces an observed real-world phenomenon. During model construction, we have a dual focus on accurately incorporating the mechanism and on devising ways to characterise its effect in a form that can be compared with data or observations. Comparisons with observations can be more or less formal depending on whether the model is highly idealised or complex, whether our predictions are qualitative of quantitative, and the form and extent of real-world observations or data. For example, formal model fitting and parameter estimation rely on the availability of data and a model at an appropriate level of complexity for the methods employed.^4^ Simple or complex models can be used to pinpoint errors in scientific hypotheses by using them to generate several predictions and establishing which are more (or less) compatible with observations. For example, in the work by Donnelly et al. (12) described in the section above, once explanations had been suggested through theory building, model predictions were used to narrow the space of possibilities. Ultimately, these authors concluded the unexpected pattern of disease incidence following culling was due to the perturbation of badger social structure and consequent movement. Additional uses of models to test theory include the use of a “null model” to serve as a baseline (or “straw man”) and sensitivity analysis to gauge the importance of model assumptions.

### Applying Theory

By applying theory, we refer to the use of models to forecast potential future events, or to predict events that might have occurred or could occur under circumstances or interventions that differ from those observed (sometimes referred to as “counterfactuals”). For example, Porphyre et al. (16) used a model to investigate the possible effects of a vaccination intervention in the event of a hypothetical introduction of FMD into Scotland, and Ferguson et al. (15) used a model to compare 2001 FMD outcomes under the implemented culling strategy with those that might have occurred without the intervention.

When motivated by the application of theory, model construction focuses on ensuring that key mechanisms are modelled as accurately as possible. Predictions are not intended to be falsified: they should be as accurate as possible so that we can compare actual and counterfactual scenarios. Steps in model use include careful construction, verification, and validation, subsequent use to conduct “experiments” under different conditions, and the examination of any effect on epidemiological outcomes (20). This use capitalises on the power of process models: to the extent that the model embodies real-world mechanisms, changing the conditions in which those mechanisms play out allows us to observe, characterise, and quantify the effects of these changes. Using models in this way requires a solid understanding of the processes acting in the epidemiological system for at least two reasons. First, empirical investigation of counterfactual scenarios is usually infeasible or impossible, e.g., in the case of historical counterfactuals, but also experimental testing of a range of policy options (also making falsification of model results impossible). Second, if models are used to inform policy decisions, incorrect predictions can be harmful, giving model use an important ethical dimension.

## Model Construction Decisions

Establishing our motivations for using process models informs lower-level modelling decisions by helping us focus on how to select and represent the parts of the system to best answer the research question. Our key decisions centre on which aspects of reality to simplify and in which ways, taking our knowledge of the epidemiological system and research questions into account. As Grassly and Fraser (18) point out, “unnecessary complexity can obscure fundamental results and is almost as undesirable as over-simplification,” thus “model choice—the process of deciding which model complexities are necessary—is a central part of mathematical modelling of infectious diseases.” In this section, we identify and discuss five important modelling decisions and associated options.

**Figure 1:**
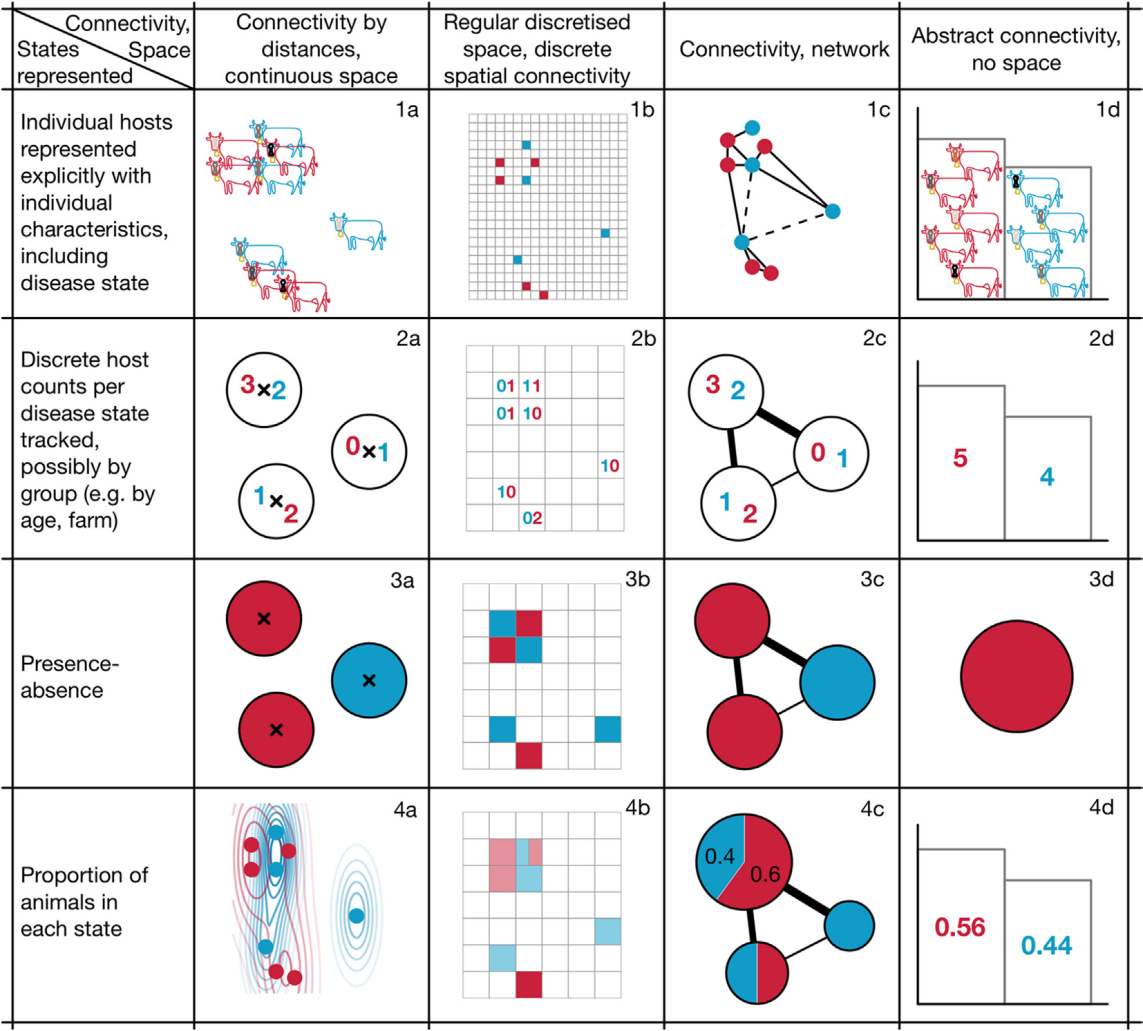
Framework of example model structures resulting from modelling decisions relating to the representation of space/connectivity and the level of aggregation/representation of states. Following convention, susceptible and infectious individuals or groups are shown in blue and red respectively.

We begin with three key decisions that apply to all infectious disease models in epidemiology: whether we want to track the infection status of individuals or groups; how to model the connectivity of hosts that determines transmission between them; and which disease states to model. These decisions relate to how we choose to represent the fundamental elements of infectious disease epidemiology: the disease states of the host population and the connections supporting transmission between them. The first two decisions determine the epidemiological states that we wish to track in the model, shown by the colours and rows of Figure 1, while different forms of connectivity are shown in the columns. Figure 1 demonstrates how their combinations can result in different model structures, while Table 2 provides descriptions of model types that include key search terms to aid with literature searching. We then describe two further decisions that relate to how we choose to implement changes of epidemiological state over time: whether these are modelled as taking place continuously or in discrete time; and whether and how to incorporate randomness into the processes that we model.

**Table 2:** Typical names used to describe the models shown in Figure 1, to assist in literature searches (particularly terms highlighted in italics); descriptions of almost all modelling approaches discussed here are provided in Ref. (21–23), as well as in the references cited throughout this article.

Figure 1 type	Usual model name/description
*1a*	Usually referred to as an *agent-based model* (ABM); sometimes an *individual-based model* (IBM). Typically, IBMs are less detailed and have fewer state variables than ABMs
	Reference for bTB (24)
*1b*	Usually referred to as a *cellular automaton* (CA) or *probabilistic cellular automaton* (PCA) if transitions between time steps are probabilistic
*1c*	*Network model*. Note that in the mathematical literature, networks are referred to more precisely as graphs and many results used in epidemiology use graph theory. Approximations to full network models include *moment closure* methods (including so-called *pairwise approximation models* or approximations based on triples, etc.)
	References for foot-and-mouth disease (FMD) (25, 26)
*1d*	Agent-based or IBM without spatial information (See 1a), possibly in the form of a *branching process* model
*2a*	Sometimes referred to as a *metapopulation model* or *patch model*, although there is some confusion in the literature regarding the distinction between these terms. (The confusion focuses on whether to refer to models that maintain individuality but of grouped individuals versus models that consider only whether a patch is occupied or unoccupied, should be referred to as patch models or metapopulations. Both are used.)
*2b*	Might be referred to as a CA or PCA (see 1b), but where each cell can contain more than one individual. Alternatively, might be referred to a *gridded metapopulation model*
	References for bTB (25, 27)
*2c*	Network model in which the network connects groups (e.g., herds) rather than individuals
	Reference for bTB (27)
*2d*	*Difference equation* model or standard *Gillespie simulation model* (also *Gillespie algorithm* or *Gillespie stochastic simulation algorithm*) in which counts of hosts in each state are integer values. Adaptations include *tau-leaping approximations*
*3a*	Usually referred to as a *metapopulation* or *patch model*. One example in continuous space is the *Spatially Realistic Levins Model*, in which patches can also have different characteristics
	Reference for FMD (28)
*3b*	CA or PCA (see 1b), where each cell is considered infectious if at least one individual is infectious (see relationship between 1b/2b and 3b)
*3c*	Network model in which each group is considered infectious if at least one individual is infectious (see relationship between 2c and 3c)
	For FMD, InterSpread (29, 30) can be used in this way when transmission is not solely a function of distance between farms
*3d*	Trivial *presence–absence model*
*4a*	*Partial differential equation* (PDE) model, *reaction-diffusion equations*
*4b*	Uncommon in the animal epidemiology literature
*4c*	Network in which the proportion of animals in each state, per network node, is modelled
*4d*	Proportion of animals in each state is modelled for a single population. Classic *ordinary differential equation* (ODE) models fit into this category
	Reference for FMD (15)

*Not all model types are used in the literature on bTB/FMD, and some, therefore, do not have a reference within this literature. Note that although much of the literature refers to only differential equation models as “compartmental models,” all models referred to in this article are compartmental models in the sense that states are discrete (an individual can only be one state, e.g., susceptible, exposed, infectious, etc.). Depending on the number of states, all could, therefore, be described by reference to the states included, so could be referred to as, e.g., susceptible-infectious-susceptible (SIS), susceptible-infectious-removed, SIR models (those in Figure 1 have only 2 states, so are SI or SIS models)*.

### How to Model Hosts

Our first decision, captured by the rows in Figure 1, concerns whether we want to track the infection status of individuals (e.g., cows) or of groups (e.g., herds). In our earlier ABM of disease spread in UK cattle (Figure 1, 1a), each cow is modelled explicitly and tracked over time, taking up its own space in computer memory. This allows us to track both the total number of infected animals and the fate of individual cows; however, as the number of cows increases, so too does the computational cost of simulation and potentially the complexity of outputs, making them difficult to interpret. As a result, detailed ABMs are rarely used to model disease spread across a system as large as the UK and are more frequently used on local scales. For example, Biek et al. (24) used an ABM (Figure 1, 1a) to explore the correspondence between likely transmission pathways identified through epidemiological modelling and data on phylogenetic relationships in the bacterial population.

When detailed ABMs are too computationally intensive or complex to analyse, we might choose to group individuals. For example, it is sometimes preferable to focus on groups, such as herds or farms, or groups defined by age, sex, or spatial distribution; in Figure 1, the difference between row one and the remaining rows represents this grouping distinction. Whether grouping is an appropriate decision depends on both the system and the research question. For example, it makes sense when individuals fall into relatively clear groups that have important implications for their epidemiology, or if we have better information about group-level than individual-level epidemiological processes.

In rows 2, 3, and 4, we track groups rather than individuals, and the distinctions between these rows relate to the kind of information we choose to track about group infection status. There are several main alternatives: we could track the number of individuals displaying each infection status (row 2), the presence (or absence) of hosts in each state (row 3), or the density or proportion of hosts in each state (row 4). Models that track the number or proportion of hosts in one of three disease states (susceptible, infectious, and removed) may be familiar as simple SIR-type transmission models based on ordinary differential equations (ODEs). A further simplification consists of tracking only the presence (and absence) of infection in groups of hosts.

In addition to increased analytic and computational tractability, summarising the infection status of a group of hosts sometimes better represents the scale at which data are available, or at which transmission processes are understood. However, one drawback of modelling groups rather than individuals is that we only know that at least one animal is infected, losing information about which individuals are infected. Grouping also forces us to aggregate, so we lose the capacity to study within-group heterogeneity. Further, if a model tracked the disease state of farms (Figure 1, 3a) rather than cows (Figure 1, 1a), the influence of individual attributes could no longer be investigated. Presence-absence and density approaches (rows 3 and 4 in Figure 1) are usually more appropriate for systems in which randomness and individual-level variation are small. However, they are more difficult to justify when we are interested in modelling processes in which individuality matters. For example, they can cause problems when we are interested in studying extinction because continuous population densities mean that the pathogen population can become arbitrarily small (e.g., less than one pathogen present) without going extinct.

**Figure 2:**
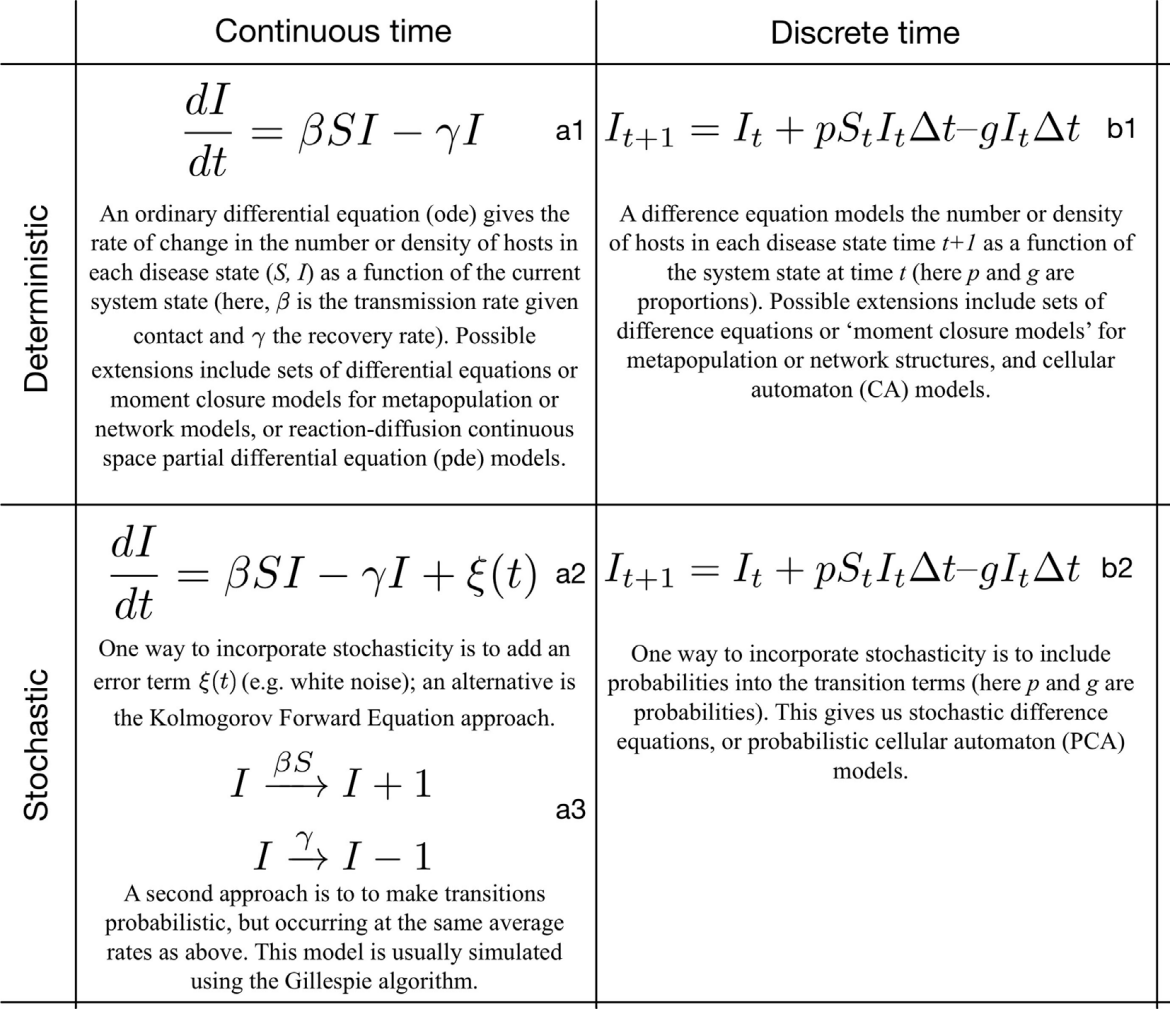
Illustrative examples of deterministic and stochastic models and their symbolic formulation for continuous and discrete time.

The grouping of cattle into herds, each of which is associated with a farm, underpins most models of FMD and bTB transmission in the UK. For example, in the InterSpread modelling framework used during the 2001 FMD outbreak in the UK, researchers initialised all UK farms with counts of the number of different kinds of livestock recorded during the most recent farm census (29, 31), and simulated whether each farm was susceptible or infected (the framework is able to capture different distance measures, so corresponds to Figure 1, 3a and 3c). Similar approaches to modelling the spread of bTB within and between farms have been implemented, tracking the numbers of animals moving between farms, and the number and disease state of animals on each farm, with some work using a combination of approaches from row 2, e.g., Ref. (27), that involves both a tiling and a network, i.e., Figure 1, 2b and 2c. In Ref. (28), only the presence or absence of infection in groups is tracked (Figure 1, 3a).

### How to Model Connectivity

Our third modelling decision, represented by columns in Figure 1, relates to how infection passes between individuals or groups of hosts. In our original ABM, hosts move in continuous space,^5^ and space itself determines host connectivity—for example, pathogen transmission might be modelled as occurring when agents are sufficiently close to one another (Figure 1, 1a), or as a continuous function of distance. Models can also represent space as continuous without explicitly representing individual hosts, e.g., by modelling the location of farms in continuous space, with distances between farms affecting the spread of infection among them (Figure 1, 2a). This is an appropriate approach when our primary interest is between-farm transmission and spatial distance is considered a good proxy for strength (or probability) of potentially infectious contact. A continuous space approach that models the proportion of infected hosts is that of reaction–diffusion models that are based on PDEs (Figure 1, 4a).

One alternative to modelling hosts in continuous space is to divide the modelled landscape into discrete areas in the form of a tiling (often referred to as a grid or lattice), as in the example scenarios shown in Figure 1 column b. A tiling can be composed of regular shapes such as hexagons or squares (as in Figure 1), as is often the case for satellite data; alternatively, it can be irregular, as might be the case for administrative jurisdictions. Discretisation of space is often used when model outputs will be compared with data that are only available in spatially discrete form, or when epidemiological interventions are necessarily applied over predetermined areas because of administrative jurisdiction. Each patch in a tiling can have different characteristics (e.g., host density), making it possible to examine the influence of this heterogeneity.

A tiling covers the full space and can, therefore, only be used when connectivity can be collapsed to two dimensions (32); however, often there are also parts of the landscape that we do not need to model explicitly. This can occur in the case where hosts aggregate in housing or pastureland, or when non-spatial mechanisms such as human-mediated transport or watercourses determine transmission. A spatial tiling is a special case of a network that takes a lattice form, and a more general network approach can be valuable for this kind of problem, modelling farms as nodes in a connectivity network, with the strength of the links between farm nodes determining the probability of transmission from one to another. Link strength could, for example, be determined by the shortest distance between farms when travelling by road, or by previous trading history between them. This broad category of network models is represented by the diagrams in Figure 1, 2c, 3c, and 4c. The approach is similar to that of metapopulation models used in ecology, in which the network connections or distance influence otherwise independently modelled populations that exist in patches.^6^ This type of model, usually represented in the form of a distance- or contact-matrix, was the basis of several FMD models in which transmission was modelled using known distance between farms, e.g., Ref. (28), making it a model of type 3a in Figure 1. Using distances between farms (Figure 1, 3a) is also a special case of Figure 1, 3c, where these distances determine the modelled connection between nodes.

The final broad category of model approaches to connectivity is represented by column d in Figure 1, in which the effects of space are not modelled. The most familiar form of disease model in which space is implicit is the simplest SI model, represented by Figure 1, 4d, in which all modelled individuals have the same probability of encountering one another per unit time, as if they occupied a theoretical homogenous space and mixed randomly within it. This “complete mixing” assumption may be appropriate for certain systems (perhaps for water-borne diseases of fish), but can also be used as a simplification when the complexity of other aspects of a model (e.g., number of disease states) make a spatially explicit model difficult to analyze. Non-spatial models can also be used to track only the presence or absence of disease in a system (Figure 1, 3d), modelling numbers of individuals (Figure 1, 2d), or keeping track of distinct individuals (Figure 1, 1d).

The decision about how to model connectivity is usually based on a combination of factors including data availability and our understanding of disease processes. For example, for the analysis of local culling policies for the 2001 FMD epidemic, one difficulty was that individual farms often contained multiple parcels of land whereas the data only represented each farm as a single spatial point. As a result, many more farms were actually contiguous (and thus needed to be subject to culling) than was apparent from the available data. However, by grouping farms into discrete tiles with neighbouring tiles used to establish contiguity and counting the number of infected farms per tile (as in Figure 1, 2b), it was possible to mimic the extent of culling recorded during the epidemic and, therefore, explore counterfactual culling policies (25). In this case, a discretisation of space allowed the simulation of more realistic interventions. Modelling in discrete space can also be used as a tool for detecting the spatial scales of key processes driving transmission, e.g., Ref. (33) for a general epidemiological example. Figure 1, 1c also highlights that it is possible to model the connections between individuals as a network, as in social network analysis models or models of sexually transmitted disease spread, or between farms *via* the movement of livestock (26).

### How to Model States

Once we have decided on a level of grouping of hosts and how infection passes between them, we need to decide what states, captured by colours and spatial locations and other attributes in Figure 1, each individual animal or group can take. In the simplest model, we might decide that each host (individual or group) can have only two states, susceptible or infectious. Classic SI models based on differential equations are models of this type. However, we might decide to include additional states such as spatial location or age, or additional disease states that capture incubating or immune status, or changes in the level of infectivity during different stages of infection.^7^ In Figure 1, colours are used to illustrate disease state, with blue and red used to represent susceptible and infected cows or patches; in 1a, differences in shading of the markings on the head of each cow emphasise that individuals have additional distinct attributes and states, while in columns a and b, the position of crosses, points, and square cells denote spatial location.

As we increase the number of attributes and states that we model, the complexity of the model grows quickly. For example, if we model space using patches/nodes or a grid, the full system state at any given time consists of the combination of the states of all the patches. For example, even for the simple presence–absence model in Figure 1, 3a, although each patch has only two states, the whole system has 2^3^ = 8 states. By using “R” to represent red and “B” for blue, these states are: (R,R,R), (R,R,B), (R,B,R), (B,R,R), (R,B,B), (B,R,B), (B,B,R), and (B,B,B). Adding a single patch leads to 2^4^ = 16 system states. As the numbers of patches or animals and states grow, the number of possible system states grows very quickly. Although we do not expect all possible system states to occur, even if only a small proportion of these arise during the dynamic process captured by the model, this number can be very large. As a result, it is often helpful to track instead a one-dimensional variable, such as the number of infectious hosts or groups in the system.

### How to Model Time

The order of events—the sequence in which farms become infected—and whether two events occur close together in time or far apart, can have important effects on disease dynamics, so determining how to model time forms an important consideration in the construction of process models.

In determining how to model the progress of time, it is helpful to distinguish between epidemiological processes that can occur at any time—i.e., in continuous time—and those in discrete time. Modelling approaches have been developed that respect this distinction between continuous and discrete time and are named accordingly. Biological processes take place in continuous time in the sense that the interval between events can be arbitrarily small, so in some senses, discrete time representations are always an approximation, with a key difference being that when time steps are sufficiently long in discrete time models, more than one event can occur at the same time. Nonetheless, if events are highly clustered in time, for events within the same time window, it may not matter—and it may not even be possible to decide—which happened first. For example, within the period of a day, it may not be important which cow became infectious first, especially if cows only come into contact during milking. In a more extreme scenario, the host population might be periodically eliminated (e.g., harvested crops), or there could be periods of the year during which new infections cannot arise (e.g., if vectors overwinter in diapause). In these cases, we have a biologically driven reason to model in discrete time. Discrete time representations can also be used as an approximation to continuous time processes, perhaps because researchers are more comfortable with the modelling techniques or in cases where discrete time approximations are less computationally expensive.

When discrete time approaches are used, it is important to choose an appropriate time step length. When discrete time modelling has a biological justification, the length of the interval should be chosen so that it can reasonably be assumed that the order of events within the same time step is unimportant. This means that we need to focus on the fastest process in the system, typically transmission dynamics rather than host demographic processes. When discrete time is used as an approximation, time needs to advance in very short steps; however, it is usually very difficult to decide how short the interval needs to be to avoid influencing model predictions. For example, Mancy et al. (34) showed that the outcome of spatial competition between two species differed between a continuous and a discrete time model, even for very short time steps. It is, thus, important to report whether a discrete or continuous time model is employed along with the rationale for the decisions made.^8^

In general, the decision about whether to model in continuous or discrete time is driven primarily by the biology of the system under study and associated research questions, rather than our motivations for modelling. Although when testing theory, time is often modelled discretely to support comparisons with data, if real-world processes are continuous, it is often preferable to model in continuous time and then aggregate model output to correspond to data intervals. Similarly, when applying theory, the timing of interventions should be modelled according to the feasibility of implementing these interventions in the real world. Examples of continuous time modelling paradigms are differential equations (both ordinary and PDEs) and simulation approaches such as the Gillespie algorithm (modelling discrete events in continuous time, Figure 2, column a),^9^ whereas discrete time approaches include difference equations and cellular automata. The symbolic form of these is shown in Figure 2 (column b).

### Whether and How to Model Stochasticity

The final decision we discuss is whether to model epidemiological processes as deterministic or stochastic. Although familiar, this distinction bears repeating as it relates to differences in both the relationship between models and the real world, and to the steps required for their use. Deterministic models are those in which outcomes are entirely predictable based on the parameter values; in contrast, the output of stochastic models is not fully dependent on parameter values so cannot be predicted precisely. This means that deterministic models only need to be solved once for each set of parameter values, whereas stochastic models need to be run multiple times to gain good insight into the “average” or “typical” outcome. Running a model multiple times creates an operational overhead; however, the variation generated allows us to exploit information on the distribution of outcomes in real world data in their validation.

The first issue that arises is whether deterministic or stochastic models provide a better representation of the system we are modelling. The discussion about whether the universe is truly deterministic or stochastic is an unresolved debate in the philosophy of science literature; however, because epidemiological systems are never fully known, it is often preferable to use stochastic models. Bolker (35) partitions stochasticity into three sources of random variability: process-related stochasticity in the form of either endogenous stochasticity or environmental stochasticity and measurement error. *Endogenous stochasticity*^10^ is variability which is inherent to the system itself and that would occur between realisations even under identical environmental (or experimental) conditions, including variability in host demographic processes and the number of secondary cases. *Environmental stochasticity* refers to the unpredictability of exogenous processes (i.e., those outside of the system of interest and that occur independently of it), such as extreme weather events that affect host demography or disease dynamics. Depending on where we locate the limit between our system and the environment, the same source of stochasticity might be thought of as endogenous or exogenous: for example, weather and climate stochasticity are usually treated as environmental; in contrast, stochasticity in individual farmer responses to policy interventions might be viewed as exogenous or as part of the system. In contrast to process stochasticity that exists regardless of whether we study the system, *measurement error* arises in conjunction with our role as scientists and refers to variability in data due to difficulties of measurement. Within this category, Clark and Bjørnstad (36) refer to measurement inaccuracy, missing data points, lags between the biological process of interest and measurable outcomes, and “hidden” system states that are not amenable to measurement; methods for dealing with measurement error are discussed in Calder et al. (37).

Starting from deterministic models in the form of, for example, ODEs or difference equations (Figure 2, a1, b1), there are several ways in which process stochasticity can be incorporated into epidemiological models. Two common approaches are the inclusion of a stochastic error term into an otherwise deterministic framework (Figure 2, a2) and the use of a fully stochastic process model (e.g., a Markov model) (Figure 2, a3, b2). In the first, epidemiological processes are modelled as having a deterministic component, with variability around this deterministic trajectory modelled as “noise.” In a fully stochastic approach, the state of the system depends on previous states and a random component, with transitions occurring according to probabilities often represented in matrix form. If we decide to ignore states further back in time, we can employ results from mathematical Markov process theory, including those that facilitate simulation approaches using the Gillespie algorithm, a continuous time, event-driven approach (38, 39). In contrast to ODE models, advantages of simulating Markov processes (e.g., using the Gillespie algorithm or discrete-time equivalents) are that negative population counts and partial individuals are not possible and it is possible to identify a precise extinction time. For the Gillespie algorithm, there is a clear relationship with deterministic ODE approaches, including *R*_0_ calculations, meaning that results from these simpler models can be compared with simulation outcomes. The Gillespie algorithm assumes exponentially distributed waiting times, which are often unrealistic (40), so it may be necessary to combine exponential distributions *via* the so-called “method of stages” (41), or associate the approach with alternative simulation algorithms for to achieve other distributions, e.g., for infectious periods.

In addition to determining the type of model to use, the decision between deterministic and stochastic models also affects the steps involved in model use. “Solving” a process model can refer to obtaining either long-run outcomes, often in the form of an equilibrium solution, or to obtaining the time path of the system states. For each set of parameters and initial conditions, solving a deterministic process model leads to a single time path. For simple deterministic systems, it is sometimes possible to solve for time paths or equilibria analytically (i.e., symbolically). For more complex systems, numerical methods that allow us to obtain approximate solutions are often available (e.g., numerical methods for solving differential equations such as Runge–Kutta and variants thereof).^11^ This means that we only require one solution of the model per parameter set (and initialisation, where appropriate). In contrast, stochastic models lead to a set of solutions and associated probabilities. For some types of stochastic models, numerical methods are available to obtain certain general results (e.g., the stationary distribution of a Markov chain can be obtained from eigenvector relations, for which numerical methods are available), and some more complex models can be solved in this way if we are interested only in summary statistics such as means. However, in many cases, it is necessary to use stochastic simulation in which system states are computed as a function of previous states and transition probabilities, and for each initialisation and parameter set, multiple solutions are obtained. We are, therefore, required to simulate multiple times for each parameter set and initialisation and compute summary statistics on model output.

When deciding whether and how to incorporate different sources of stochasticity into process models, it is helpful to consider the system, the questions we want to answer and our motivations for using a model. In relation to the system, a commonly recognised point is that stochasticity causing random population size fluctuations has stronger effects in smaller systems. In disease ecology, stochasticity is more important when the host population is small, but also at the very beginning and end of an outbreak when there are fewest infectious agents (18). Stochasticity is also important for certain questions: for example, process stochasticity is important when studying pathogen elimination (39), not just because pathogen populations are small close to elimination but also because populations can go extinct through random processes even when their deterministic equivalent persists. Further, incorporating existing knowledge about stochasticity in epidemiological processes can be intrinsic to certain questions: for example, O’Hare et al. (14) used knowledge of variability in the number of secondary cases to guide model choice decisions when investigating the role of “superspreaders.” The type of answer required can also drive decisions. For example, mathematical tools for deterministic models are also generally more developed, meaning that it is possible to obtain analytic expressions or precise numerical estimates of quantities of interest, and is easier to examine threshold behaviours. In such cases, choosing between deterministic and stochastic models is, therefore, driven primarily by our questions, rather than our motivations. Nonetheless, especially when exploring theory, we may decide to ignore stochasticity altogether or include only one form at a time, because this allows us to isolate the effect of each in conjunction with parameter changes. Incorporating measurement error is important when the motivation for using models involves using or explaining data or observations affected by measurement error; when using models for exploring theory, it can often be ignored.

## Applying the Model Construction Approach

To apply the approach outlined here, we would begin by identifying our motivations for model use to guide us towards the aspects of model construction that require the most attention. We would then identify the biological entities that need to be distinguished, and then consider whether and how each of these should be modelled, according to the dimensions discussed above, referring to the references provided and standard texts on process modelling. This approach can also be applied when analysing the relationship between studies reported in the literature, to compare and contrast model-based findings. This should make it easier to pinpoint complementarities between approaches used to address very similar questions about related (or even the same) epidemiological systems. Indeed, although the examples provided in this article all relate to bTB or FMD in cattle, different modelling decisions are made in different pieces of work, as was also the case for work focusing on the 2001 FMD epidemic (42). For example, Ferguson et al. (15) investigated the potential for exploiting local clustering of transmission to target culling, and chose an approach (deterministic moment closure) that formulated disease spread in the context of an ODE model (as in Figure 1, 4d) but where individual states represent not just the status of individual farms, but the combined statuses of triplets of farms (e.g., not just states S, I, and R in an SIR model, but with S-S-I an explicit state representing the proportion of triplets with two susceptible farms and one infected farm). Geographical space was represented abstractly by “counting,” on the map of farms in Great Britain, the proportion of times two neighbouring farms shared a neighbour (this proportion is commonly called the “clustering coefficient”). This decision may have been motivated both by their previous analytical approaches and the need to provide rapid, responsive advice. However, similar situations can lead to different decisions: Keeling et al. (28) had previously used deterministic moment closure models to describe epidemiological invasions scenarios, but in 2001 developed a stochastic “transmission kernel” simulation approach (as in Figure 1, 3a with farms as the individuals and transmission probability declining with distance) in order to capture the explicit heterogeneity in transmission potential of FMD across Great Britain (28). Our examples are drawn from research on bTB and FMD, but for other ecological or epidemiological systems, different sets of model types might be more appropriate. For example, for diseases of wildlife, natural host groupings corresponding to herds may not exist, making this simplification inappropriate, or host movements or demographic processes might be highly seasonal, leading to different decisions about how to model time. In addition, because bTB and FMD are reportable diseases in the UK, good datasets exist for their tracking; however, for other diseases or in areas of the world where this is not the case, more limited data can influence modelling decisions.

Once possible modelling options are identified, their appropriateness can then be (re)considered with reference to the epidemiological system, our motivation for using modelling, and the precise questions asked. It is therefore valuable to identify explicit criteria for assessing whether modelling decisions are satisfactory in terms of their accuracy and level of detail. Drawing on military terminology, Holling (43) contrasted *strategic* models, usually designed to be as simple as possible to reveal potential explanatory generalities, and *tactical* models, deliberately higher in complexity because they are designed to predict the dynamics of specific systems. Historically, models used to explore theory have typically been simpler than those used to apply it, in part because they have been more amenable to analysis using mathematical techniques. However, contemporary techniques including principled use of computer simulation (44) and mathematical tools for the analysis of stochastic systems (45) have made it easier to conduct theory exploration and obtain general results, even for relatively complex models. Although still a common heuristic, the distinction between simple and complex models and their relative roles in relation to motivations for using models is beginning to break down (46).

In determining the appropriate level of complexity or output accuracy, we focus on three factors: the respects, the degree, and the specificity of the system to which results should apply (5). When we determine the *respects* in which a model is required to be accurate or sufficiently detailed, we are asking the qualitative question “what epidemiologically relevant phenomena do we want our model to reproduce?” For example, when using models to explore theory, we may decide that it is sufficient that our model provides information on whether a disease persists or goes extinct; however, when we use models to apply theory, we may also want the model to provide information on time until extinction or the spatial locations at which a pathogen is likely to persist the longest. When we determine the *extent* to which our model is required to be accurate, we ask the quantitative question of “how close do we need those phenomena to be to those seen in the real world?” In terms of model outputs, this question is primarily relevant when we use models to help us generate, test and apply theory. However, in relation to model inputs, such as parameter values and initial conditions, it can apply to all motivations for model use. When we determine the *system* for which our model is required to be sufficiently accurate or detailed, we are asking “for what systems do we want this to apply?” For example, much of the more theoretical work using process models, often in the form of theory exploration, is deliberately general and a specific disease system may not be mentioned (e.g., it may apply to abstract SIR processes); when applying theory, it is usually critical for the model to relate to a specific disease system, but it may be sufficient for it to be accurate for a country or region, or host breed. Decisions about the level of detail or accuracy required of a model will often be driven by practical considerations such as funding, publication targets, or data availability.

## Discussion

Historically, the majority of veterinary disease modelling has followed a statistical approach, in which the focus has been on characterising statistical associations between a response variable and explanatory variables. For example, we might use this approach to identify farm factors—e.g., sanitation practices—that affect risk of an outbreak. This form of modelling becomes increasingly involved if the explanatory variables interact with one another, or if the response variable depends on its previous values. Although techniques have been developed to cope with interactions between the variables of interest, these become increasingly unwieldy as the number of interactions increases. Further, even the sophisticated techniques developed to account for these interactions usually only identify them as statistical relationships and do not explicitly represent the direction of causality in the links between them. Furthermore, models of this type are particularly reliant on all variables being available within a single dataset, using information on measurable states to infer knowledge of processes from interactions between variables that define those states. Process models, on the other hand, focus explicitly on biological processes. They can, therefore, generate considerably greater insight for investigations of the impact of changes to those processes, for example, to understand the population level impact of imperfect vaccination. Recent developments include the introduction of Bayesian modelling approaches, such as hidden Markov models that are underpinned by process models, and that bridge statistical and process approaches.

There are good reasons for increasing interest in process modelling among disease ecologists and veterinary epidemiologists. Yet, making decisions about how to construct process models, and knowing how to compare different approaches in the literature, is complicated by the existence of multiple process model types and the difficulty of establishing the relationship between them. In applied work, the model is often treated as a tool, so only the chosen modelling approach is described without comparison with alternatives, while in more theoretical work focused on developing new modelling approaches, space often limits comparative discussion to a small set of related modelling approaches. Further, most introductory texts on process modelling in disease ecology proceed by describing prototypical examples of a small range of modelling paradigms. This tends to obscure the relationships among modelling approaches and fails to make explicit the link between the appropriateness of different approaches for different systems, while reinforcing the popularity of particular paradigmatic approaches somewhat arbitrarily.

Ideally, model construction decisions should be guided primarily by the system, what we know about it, and our scientific questions. Nonetheless, our decisions are often constrained to some extent by practical considerations, including technical limitations (e.g., computational resources) and modelling knowledge. Although the increasing development of specialist software simplifies the mechanics of modelling, understanding the modelling assumptions embedded within any software used is important for accurate interpretation of outputs. As models incorporate more and more components—such as in the case of ABMs or complex models represented in matrix form—we can quickly reach a situation in which more information needs to be available to simulate or solve the model than can be held in computer memory. Time can also be a constraint if models take a long time to run or solve, especially if they need to be run or solved multiple times. Depending on which aspects of the outputs we choose to store, these can put a strain on storage capacity, and although hard disk storage capacity is increasingly cheap, managing these large volumes of data, both in terms of transferring data between devices and maintaining a file structure that is easy to navigate, can be challenging. In relation to technical knowledge, there are limitations to the number of techniques we can acquire, and it is often preferable to sacrifice some accuracy or detail in modelling decisions to allow us to use an approach that we understand well, in terms of its strengths, weaknesses, and underpinning assumptions. A strong understanding allows us to safeguard against known pitfalls, and critically, to better account for any assumptions in interpreting model outcomes. In interdisciplinary work or work at the research–policy interface where team members often have different skillsets, it can also be advantageous to use a form of modelling that all team members can understand.

In this article, we have described one way to approach model construction, based around a set of modelling decisions and their relationship with the system under study, the research questions, and our motivations in using modelling. In most cases, we have presented modelling decisions as though they were either/or decisions. In reality, within the same project, several motivations might underpin model use. Similarly, researchers often use several models, and do so with a range of motivations. For example, we might begin by using a modelling exercise to formalise our ideas about an epidemiological system, constructing a model that we use to explore theory, ultimately using it to test theory once we have acquired appropriate data (47). We might also try out multiple modelling frameworks in a single piece of work. For example, it is often very valuable to begin with a relatively simple model that we understand well or that has been analysed previously and incrementally add or change the epidemiological processes involved. This allows us to understand the effects of these changes, as well as to locate any errors in our logic or solution processes. For example, even if we are interested in the effects of host population heterogeneity, we often begin with a simple model of a well-mixed host population for comparison. Indeed, using multiple model types to address the same problem is often very useful, both within and between research teams, as redundancy, overlap, and replication serve to reduce the risk of unidentified errors (48).

To conclude, we believe that the structured approach presented here, based on the identification and classification of model construction decisions, should help those new to epidemiological modelling to reach a level of model construction expertise more quickly, while providing an analytic structure and terminology for more experienced readers. This conceptual analysis helps clarify the relationship between the biological system and the assumptions about it embedded in the model and highlights the similarities and differences between modelling approaches.

## Author Contributions

RM and PB developed the conceptual framework for model type analysis, planned, and drafted the manuscript. RM produced the figures. RK contributed scientific content and to manuscript revision. All authors have critically reviewed and revised the manuscript and approved the final product.

## Conflict of Interest Statement

The authors declare that the research was conducted in the absence of any commercial or financial relationships that could be construed as a potential conflict of interest.

## Footnotes

^1^The relationship between model and biological mechanism differs between process models and statistical approaches based on linear models, traditionally the mainstay of research and education in biology. Although these statistical approaches are often guided by mechanistic theory and employed with the aim of understanding biological mechanisms, the mechanisms themselves are not modelled directly and are instead inferred from associations with explanatory and response variables. In contrast, process models incorporate processes explicitly based on biological understanding, potentially in the absence of detailed data on every aspect of the modelled system. They can be thought of as simplified “model worlds” in which key epidemiological processes unfold over time, analogously to their progression in the real world.

^2^Despite this restriction, much of the conceptual ground covered in this article applies to any biological discipline in which process models are used as research tools (e.g., ecology). We return to this point in the Section “Discussion.”

^3^Although motivations for modelling have been classified in different ways, see, e.g., Ref. (8), we draw on the five categories described by Odenbaugh (5). We build on these by clarifying distinctions between them, adapting them to the context of epidemiological process modelling, and clearly identifying the steps in model construction and use associated with each. We rename certain categories for conceptual clarity: Odenbaugh refers to Mapping and formalising theory as “providing conceptual frameworks,” to Building theory as “generating explanations,” and to a category similar to Testing theory as “investigating more complex systems.”

^4^A full discussion of formal model fitting and parameter estimation is beyond the scope of this paper, but this is an area of particularly active development, see, e.g., discussion in Ref. (19).

^5^When modelling in continuous space, each host has an (x,y) location, in which x and y are not restricted to integer values (i.e., can have arbitrarily many decimal places).

^6^The emphasis of heterogeneity in metapopulation models is typically on the attributes of patches, and patches are usually thought of as being equally connected or connected by distance. In network models, it is more common to assume nodes are homogeneous and emphasise different levels or kinds of connectivity between them. Nonetheless, this is a question of research tradition and emphasis, and a model from one of the two frameworks can be thought of from either perspective.

^7^States could be either discrete (as in compartmental models) or continuous (e.g., antibody titer).

^8^It is not uncommon to read articles in which this information is not provided. One tip to determine whether a model represents time as continuous as discrete is to search for the keyword “time step” or “timestep” in the text and figure captions.

^9^Although, in the Gillespie algorithm, time between events is “skipped over” such that simulation time progresses in a step-wise manner, it is nonetheless a continuous time approach because each step can have arbitrarily many decimal places (i.e., is a real number, to the limits of computational accuracy).

^10^Bolker refers to endogenous stochasticity as demographic stochasticity; however, the term is awkward in infectious disease epidemiology in which both host and pathogen demographic processes are important.

^11^Although numerical solutions are often referred to as simulations, it is useful to distinguish between the two. A simulation is defined as the imitation of a process over time. In a stochastic simulation such as an ABM/IBM, the computer simulation is an imitation of real-world processes, but is the actual playing out of the model. In contrast, a numerical algorithm used to solve a differential equation model imitates the differential equation; strictly speaking it is, therefore, not a simulation of the real world processes (although it is a simulation of the differential equation model).
